# Preliminary study on prevalence of hemoprotozoan parasites harbored by *Stomoxys* (Diptera: Muscidae) and tabanid flies (Diptera: Tabanidae) in horse farms in Nakhon Si Thammarat province, Southern Thailand

**DOI:** 10.14202/vetworld.2023.2128-2134

**Published:** 2023-10-18

**Authors:** Tanakorn Phetkarl, Punpichaya Fungwithaya, Supak Udompornprasith, Jens Amendt, Narin Sontigun

**Affiliations:** 1 Akkhraratchakumari Veterinary College, Walailak University, Nakhon Si Thammarat, 80160, Thailand; 2 School of Agricultural Technology, King Mongkut’s Institute of Technology Ladkrabang, Bangkok, 10520, Thailand; 3 Institute of Legal Medicine, University Hospital Frankfurt, Goethe-University, Kennedyallee 104, 60596, Frankfurt am Main, Germany; 4 One Health Research Center, Walailak University, Nakhon Si Thammarat, 80160, Thailand; 5 Center of Excellence Research for Melioidosis and Microorganisms, Walailak University, Nakhon Si Thammarat, 80160, Thailand

**Keywords:** hemoprotozoan parasites, *Stomoxys* flies, tabanid flies, Thailand, vector

## Abstract

**Background and Aim:**

*Stomoxys* and tabanid flies are of medical and veterinary importance because they play crucial roles in disease transmission as mechanical vectors of various hemopathogens. However, its role as a hemoprotozoan parasite vector in horse farms has not been studied. Therefore, we investigated the occurrence of hemoprotozoan parasites belonging to the genera *Babesia*, *Theileria*, and *Trypanosoma* in *Stomoxys* and tabanid flies using conventional polymerase chain reaction (PCR) and DNA sequencing.

**Materials and Methods:**

All samples (*Stomoxys* and tabanid flies) were collected using an Nzi trap for three consecutive days each month from November 2022 to March 2023. The flies were morphologically identified to the species level and separated according to sex. Individual (for tabanid flies) or pooled samples (consisting of three specimens of *Stomoxys* flies of the same species and sex collected from the same site) were used for DNA extraction. Conventional PCR was used to screen for hemoprotozoan parasite DNA, followed by Sanger sequencing to identify the species.

**Results:**

In total, 189 biting flies were collected, including four species of *Stomoxys* (*Stomoxys bengalensis*, *Stomoxys calcitrans*, *Stomoxys indicus*, and *Stomoxys sitiens*) and five species of tabanids (*Atylotus cryptotaxis*, *Chrysops dispar*, *Tabanus megalops*, *Tabanus mesogaeus*, and *Tabanus rubidus*). *Stomoxys calcitrans* was the most prevalent species, accounting for 58.7% (n = 111) of the collected flies. Ten (12.4%) of the 81 samples (individuals and pools) analyzed by PCR were positive for the 18S rRNA gene of the *Theileria*/*Babesia* species. *Trypanosoma* DNA was not detected in any sample. After performing Basic Local Alignment Search Tool searches and a phylogenetic analysis, only six samples (7.4%), including *S. calcitrans* (n = 2), *S. sitiens* (n = 2), *T. megalops* (n = 1), and *A. cryptotaxis* (n = 1), were found to be infected with *Theileria sinensis*. Furthermore, apicomplexan parasites, namely, *Mattesia* spp. and *Colpodella* spp., were found on *S. indicus*, the fungus *Meira* spp. was found on *S. calcitrans*, and the pathogenic green alga *Helicosporidium* spp. was found on *A. cryptotaxis*.

**Conclusion:**

This study is the first to report a variety of *Stomoxys* and tabanid flies collected from horse farms in Thailand, which were found to be infected with *Theileria* and *Colpodella* species that affect mammals, suggesting that *Stomoxys* and tabanid flies can be used to confirm the presence of hemoprotozoan parasites in the study area. Understanding the presence of hemoprotozoa in flies could help design vector control programs and manage various diseases in the study area.

## Introduction

*Stomoxys* flies (Diptera: Muscidae) and tabanid flies (Diptera: Tabanidae) are obligate blood-sucking insects. Both sexes of *Stomoxys* flies and the females of tabanid flies of most species are capable of biting and feeding on a wide variety of animals (e.g., cattle, horses, dogs, goats, and wild animals), causing annoyance, irritation, skin lesions, and blood loss, causing significant health problems and decrease livestock productivity. Furthermore, they are mechanical vectors for several pathogens, including viruses, bacteria, protozoans, and helminths [1–5]. Some species act as intermediate hosts for certain nematodes (e.g., *Loa loa*, *Habronema microstoma*, and *Dirofilaria repens*) [6, 7] and as biological vectors of *Trypanosoma* (*Megatrypanum*) *theileri* [2, 8]. Therefore, *Stomoxys* and tabanid flies are of medical and veterinary importance.

The genera *Babesia*, *Theileria*, and *Trypanosoma* are major hemoprotozoan parasites that cause babesiosis, theileriosis, and trypanosomiasis, respectively, in wild and domestic animals (e.g., cattle, goats, and horses), particularly in tropical and subtropical areas [9–12]. Most of the parasites, except for *Trypanosoma* spp., are biologically transmitted by ixodid ticks, but they can also be mechanically transmitted by *Stomoxys* and tabanid flies [1–5].

In Thailand, the species diversity and seasonal abundance of *Stomoxys* and tabanid flies have been reported in several habitats, such as beef cattle farms, buffalo farms, and national parks [13, 14]; however, little is known about the prevalence of these flies in horse farms [15]. Hemoprotozoan parasites have been frequently investigated in host animals (e.g., cattle, buffaloes, goats, and horses) and in major tick vectors [9, 11, 16, 17]. Although the role of *Stomoxys* and tabanid flies as disease vectors for animals and humans has been studied, only a few studies have documented hemoprotozoan parasites in these flies in Thailand [18–20].

This study investigated the occurrence of hemoprotozoan parasites belonging to the genera *Babesia*, *Theileria*, and *Trypanosoma* in *Stomoxys* and tabanid flies in horse farms.

## Materials and Methods

### Ethical approval

This study was approved by the Institutional Animal Care and Use Committee of Walailak University (Approval number: WU-ACUC-65068).

### Study period and location

The study was conducted from November 2022 to March 2023. Adult *Stomoxys* and tabanid flies were collected from two horse farms (horse farm 1: N 8° 27′ 06.8076″, E 99° 57′ 11.1276″; horse farm 2: N 8° 26′ 57.2928″, E 100° 00′ 43.3656″) located in the Mueang Nakhon Si Thammarat district, Nakhon Si Thammarat Province, southern Thailand.

### Fly collection and species identification

An Nzi trap [21] was used to collect and sample adult biting flies from both farms. Flies were collected from 6 AM to 6 PM for three consecutive days each month. Flies collected each day were transported to the Parasitology Laboratory at the Akkhraratchakumari Veterinary College, Walailak University, for species identification.

In the laboratory, all collected flies were frozen for 15 min at −40°C. Only biting flies, including *Stomoxys* and tabanid flies, were morphologically identified to the species level using taxonomic keys [22–24], sexed, and counted under a stereomicroscope (Olympus, Tokyo, Japan). The identified specimens were kept individually in a 1.5 mL microcentrifuge tube containing 95% ethanol and stored at –40°C for further molecular analysis.

### DNA extraction and PCR amplification

The ethanol-preserved specimens were air-dried, and genomic DNA was extracted from a combination of three body parts (the head with mouthparts, the thorax, and the abdomen) of individual specimens (tabanid flies) or pooled specimens (three specimens of *Stomoxys* flies) using the E.Z.N.A.^®^ Tissue DNA Kit (Omega Bio-Tek, Norcross, GA, USA) according to the manufacturer’s instructions. For *Stomoxys*, three specimens of the same species and sex collected from the same collection site were pooled before extracting their genomic DNA. The concentration of extracted DNA was measured using a Nano-Drop™ One C Microvolume UV-visible spectrophotometer (Thermo Fisher Scientific, Waltham, MA, USA) and then stored at −20°C until further analysis.

Conventional PCR was used to detect hemoprotozoan DNA in *Stomoxys* and tabanid flies. The primers used in this study are listed in Table-1 [25, 26]. Polymerase chain reactions were performed in a total volume of 12.5 μL containing 2 × 6.25 μL DreamTaq Green Master Mix (Thermo Scientific, Vilnius, Lithuania), 1–2 μL DNA template (100–200 ng/μL), 0.5 μL primers at 0.4 μM for *Babesia* and *Theileria* spp., and 1.25 μL primers at 1 μM for *Trypanosoma* spp. (Table-1) and nuclease-free water to a final volume of 12.5 μL. Polymerase chain reaction was performed using a Mastercycler Pro S (Eppendorf AG, Hamburg, Germany). The PCR cycle consisted of an initial denaturation step at 95°C for 3 min followed by 35 cycles of denaturation at 95°C for 30 s. Annealing temperatures were 58°C for *Trypanosoma* spp. and 68°C for *Theileria/Babesia* for 30 s. An initial extension step was performed at 72°C for 1 min, and a final extension was performed at 72°C for 5 min. For each assay, genomic DNA samples from known hemoprotozoa were used as positive controls, and nuclease-free water was used as a negative control. The PCR products were electrophoretically separated on a 1.5% agarose gel in 1× Tris-acetate EDTA buffer, stained with SERVA DNA Stain G (SERVA, Heidelberg, Germany), and visualized under UV light using the ChemiDoc™ Imaging System (Bio-Rad, Hercules, CA, USA).

**Table-1:** Primers used for detecting hemoprotozoan parasites.

Hemoprotozoa	Target gene	Primer name	Sequences (5×–3×)	Product size (bp)	Reference
*Babesia/Theileria*	18S rRNA	Ba/ThF Ba/ThR	CCAATCCTGACACAGGGAGGTAGTGACA CCCCAGAACCCAAAGACTTTGATTTCTCTCAAG	619	[25]
*Trypanosoma*spp.	ITS1	ITS1-CF ITS1-BR	CCGGAAGTTCACCGATATTG TTGCTGCGTTCTTCAACGAA	250–710	[26]

### DNA sequencing and phylogenetic analysis

The PCR-positive samples were purified using the E.Z.N.A.^®^ Cycle Pure Kit (Omega Bio-Tek) according to the manufacturer’s instructions and subsequently sent to U2Bio Sequencing Service Co., Ltd. (Bangkok, Thailand) for Sanger sequencing using the same primers as those used for PCR. The obtained sequences were manually edited and assembled into complete bidirectional consensus sequences using the BioEdit software (version 7.2.5) [27]. All sequencing results were compared with sequences available in the GenBank database using the Basic Local Alignment Search Tool (BLAST; https://blast.ncbi.nlm.nih.gov) program for species identification. Furthermore, the maximum likelihood (ML) tree was reconstructed using Tamura-Nei [28] with a gamma distribution model and tested using 1000 bootstrap replications in MEGAX software (version 10.2.6) [29] to check the genetic relatedness between *Theileria* spp. isolates in Thailand with those from other regions of the world and other closely related species stored in genetic databases.

### Nucleotide sequence accession numbers

All sequences obtained in this study were deposited in GenBank under the following accession numbers: OQ818635–OQ818640 for *Theileria sinensis*, OQ818641 for *Mattesia geminata*, OQ818642 for *Colpodella* spp., OQ818643 for *Meira* spp., and OQ818644 for *Helicosporidium* spp.

## Results

### Diversity of *Stomoxys* and tabanid flies

A total of 189 biting flies were collected, belonging to four species of *Stomoxys* (*Stomoxys bengalensis*, *Stomoxys calcitrans*, *Stomoxys indicus*, and *Stomoxys sitiens*) and five species of tabanids (*Atylotus cryptotaxis, Chrysops dispar*, *Tabanus megalops*, *Tabanus mesogaeus*, and *Tabanus rubidus*) (Table-2). The most abundant species was *S. calcitrans* (58.7%, n = 111), and the least abundant species were *S. bengalensis* (0.5%, n = 1), *C. dispar* (0.5%, n = 1), and *T. rubidus* (0.5%, n = 1). Other species included *T. megalops* (12.7%, n = 24), *S. indicus* (11.1%, n = 21), *S. sitiens* (10.1%, n = 19), *T. mesogaeus* (3.2%, n = 6), and *A. cryptotaxis* (2.7%, n = 5). The details of *Stomoxys* and tabanid flies found at each horse farm are shown in Table-2.

**Table-2:** *Stomoxys* and tabanid flies collected from two horse farms in Nakhon Si Thammarat from November 2022 to March 2023.

Species	Study sites		Total number (%)

	Horse farm 1	Horse farm 2	
Stomoxyinae flies			
*S. bengalensis*	1	-	1 (0.5)
*S. calcitrans*	95	16	111 (58.7)
*S. indicus*	21	-	21 (11.1)
*S. sitiens*	18	1	19 (10.1)
Tabanid flies			
*A. cryptotaxis*	-	5	5 (2.7)
*C. dispar*	1	-	1 (0.5)
*T. megalops*	12	12	24 (12.7)
*T. rubidus*	-	1	1 (0.5)
*T. mesogaeus*	4	2	6 (3.2)
Total (%)	152 (80.4)	37 (19.6)	189 (100)

*S. bengalensis*=*Stomoxys bengalensis,S. calcitrans*=*Stomoxys calcitrans, S. indicus*=*Stomoxys indicus, S. sitiens*=*Stomoxys sitiens,A. cryptotaxis*=*Atylotus cryptotaxis, C. dispar*=*Chrysops dispar, T. megalops*=*Tabanus megalops ,T. rubidus*=*Tabanus rubidus, T. mesogaeus*=*Tabanus mesogaeus*

### Hemoprotozoan detection

Among the 81 samples analyzed by PCR (44 pooled samples of *Stomoxys* flies and 37 individual samples of tabanid flies), only 10 samples were positive using Ba/ThF and Ba/ThR primers targeting the 18S rRNA of piroplasmids (*Babesia*/*Theileria*) (Table-3), whereas *Trypanosoma* DNA was undetectable. After performing BLAST searches, six samples (7.4%) were identified as *Theileria* spp. (AB000270) with 99.02–100% similarity, while another four samples were identified as *M. geminata* (AY334568) with 90.94%, *Colpodella tetrahymenae* (MH208619) with 89.46%, *Meira argovae* (NG_065629) with 99.85%, and *Helicosporidium* spp. (JN869301) with 97.40% (Tables-3 and 4).

**Table-3:** Prevalance of hemoprotozoa in biting flies collected from two horse farms.

Hemoprotozoa	Horse farm 1 (n = 57)		Horse farm 2 (n = 24)		Total prevalence (%)
	
	No. of positive samples	Prevalence	No. of positive samples	Prevalence	
*Theileria*spp.	2	3.5%	4	16.7%	6 (7.4%)
*Trypanosoma*spp.	0	0.0%	0	0.0%	0 (0.0%)

**Table-4:** Results of DNA sequencing indicating the detected hemoprotozoan species in the biting flies according to collection site and sex.

Study sites	Biting fly species	Voucher code	Sex	Result of sequencing		GenBank ID	Country

				Species from BLAST top hit	% identity to BLAST top hit		
Horse farm 1	*S. calcitrans*	H1SCM34	Male	*M. argovae*	99.85	NG_065629	China
	*S. calcitrans*	H1SCM35	Male	*Theileria*spp.	100.00	AB000270	Thailand
	*S. indicus*	H1SIF62	Female	*M. geminata*	90.94	AY334568	USA
	*S. indicus*	H1SIF65	Female	*C. tetrahymenae*	89.46	MH208619	China
	*S. sitiens*	H1SSF73	Female	*Theileria*spp.	100.00	AB000270	Thailand
Horse farm 2	*T. megalops*	H2TMF1	Female	*Theileria*spp.	99.35	AB000270	Thailand
	*A. cryptotaxis*	H2ACF9	Female	*Theileria*spp.	100.00	AB000270	Thailand
	*S. calcitrans*	H2SCM15	Male	*Theileria*spp.	99.67	AB000270	Thailand
	*S. sitiens*	H2SSF16	Female	*Theileria*spp.	99.02	AB000270	Thailand
	*A. cryptotaxis*	H2ACF19	Female	*Helicosporidium*spp.	97.40	JN869301	USA

*S. calcitrans*=*Stomoxys calcitrans, S. indicus*=*Stomoxys indicus, S. sitiens*=*Stomoxys sitiens,* *A. cryptotaxis*=*Atylotus cryptotaxis, T. megalops*=*Tabanus megalops, C. tetrahymenae*=*Colpodella tetrahymenae,* *M. geminate*=*Mattesia geminate, M. argovae*=*Meira argovae*, BLAST=Basic Local Alignment Search Tool

**Figure-1:**
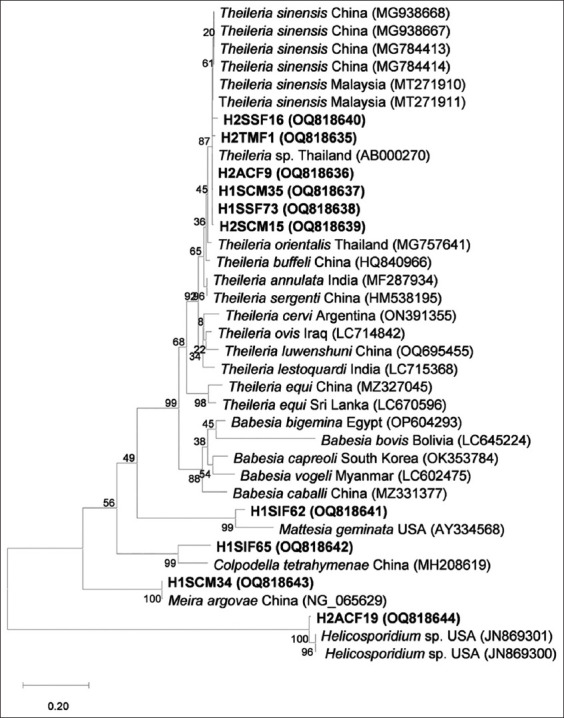
Maximum likelihood tree of 18S rRNA based on the Tamura-Nei with Gamma distribution model. Boldface letters indicate sequences acquired in this study, which include the voucher code and GenBank accession number. All reference sequences display the species name, country, and GenBank accession number. The scale bar of 0.20 indicates the evolutionary distance divergence.

The phylogenetic tree showed that the six *Theileria* spp. sequences were placed with the type Thung Song from Thailand (AB000270) and appeared in the same clade as *T. sinensis* from Malaysia and China, which was clearly separated from the other *Theileria* and *Babesia* species (Figure-1). Furthermore, the ML tree revealed that the phylogenetic positions of the H1SIF62 and H1SIF65 sequences, which were identified as *M. geminata* and *C. tetrahymenae*, respectively, from BLAST searches, fell within sister groups of the apicomplexan clade, including *Theileria* spp. and *Babesia* spp., with bootstrap values of 49% and 56%, respectively.

## Discussion

To the best of our knowledge, this is the first study on the species composition of *Stomoxys* and tabanid flies in horse farms in Nakhon Si Thammarat Province, southern Thailand, and the detection of *T. sinensis* and *Colpodella* DNA in flies collected from horse farms. In the present study, four species of *Stomoxys* (*S. bengalensis*, *S. calcitrans*, *S. indicus*, and *S. sitiens*) and five tabanid fly species (*A. cryptotaxis, C. dispar*, *T. megalops*, *T. mesogaeus*, and *T. rubidus*) were identified, of which *S. calcitrans* was abundant. High numbers of trapped *S. calcitrans*, a cosmopolitan species, were similar to the findings of the previous reports from several areas of Thailand, such as zoos, dairy farms, beef cattle farms, and national parks [14, 30, 31].

All *Theileria* 18S rRNA gene sequences in this study shared a high degree of similarity with *Theileria* spp. type Thung Song from Thailand by BLAST top hit searches (99.02%–100%); however, they formed the same cluster with *T. sinensis* sequences that were isolated from China and Malaysia in the phylogenetic analysis. These findings indicated that all *Theileria* sequences obtained in this study and *Theileria* spp. type Thung Song were *T. sinensis*. *Theileria sinensis* is classified as a benign *Theileria* species responsible for bovine theileriosis and causes benign infections in large ruminants [32]. It has been recently reported in China, Malaysia, and Russia [33–35]. In this study, *T. sinensis* DNA was detected in *S. calcitrans*, *S. sitiens*, *A. cryptotaxis*, and *T. megalops*. The presence of *T. sinensis* DNA in *T. megalops* is consistent with the previous studies, where *T. sinensis* was detected in *T. megalops* collected from beef cattle farms in the Nakhon Pathom province, central Thailand [19] and in bullfighting cattle from southern Thailand [11]. The detection of *T. sinensis* DNA in *Stomoxys* and tabanid flies indicated the circulation and maintenance of these hemoprotozoan parasites in their respective hosts in the studied areas.

Unexpectedly, the Ba/ThF and Ba/ThR primers used in this study amplified sequences that did not closely match any characterized *Theileria* or *Babesia* species. Instead, the BLAST analyzes of the H1SIF62 and H1SIF65 sequences closely matched *M. geminata* (90.94% identity) and *C. tetrahymenae* (89.46% identity), respectively, which are members of the genus that are closely related to Apicomplexa. The ML tree confirmed the relationship between these parasites and the apicomplexan lineage, as the H1SIF62 and H1SIF65, *M. geminata* (AY334568), and *C. tetrahymenae* (MH208619) sequences fell within the sister groups of the apicomplexan clade, including *Theileria* spp. and *Babesia* spp., and were located at the base of Apicomplexa in the phylogenetic tree [36]. Furthermore, these primers amplified the H1SCM34 and H2ACF19 sequences that closely matched with the *M. argovae* fungus (99.85% identity) and the pathogenic green alga *Helicosporidium* spp. (97.40% identity), respectively, according to the BLAST analyses. This suggests that these primers should be used with caution when studying the presence of *Theileria* and *Babesia* species in biting flies in the absence of sequence confirmation.

Interestingly, the DNA amplicon sequenced from *S. indicus* was found to have a high homology with that of *C. tetrahymenae* (89.46% identity), a genus closely related to the phylum Apicomplexa. Only two cases of *Colpodella* spp. infection in humans have recently been reported in China, suggesting that opportunistic infections with these parasites might occur in humans [37, 38]. Furthermore, *Colpodella* spp. have been identified in various mammals (e.g., cattle, Amur tigers, and horses), ticks (e.g., *Rhipicephalus* spp., *Ixodes persulcatus*, *Haemaphysalis* spp., and *Dermacentor* spp.), and water samples [39–41]. The presence of *Colpodella* spp. isolated from *S. indicus* in this study suggests the possible prevalence of these parasites in horse farms. This is in line with the detection of *Colpodella* spp. in horse blood [41], which revealed that horses are at major risk for becoming a reservoir for this zoonotic parasite, *Colpodella* spp., and related infections in humans. Although there have been no recorded deaths in humans or animals due to these parasites, further screening of animals, ticks, and biting flies that may harbor *Colpodella* spp. is recommended to confirm their possible presence.

The absence of typical horse parasites, namely, *Theileria equi*, *Babesia caballi*, and *Trypanosoma evansi*, in the analyzed flies suggests their absence in the studied horse farms. However, this study had limitations due to the limited number of traps and collection sites, the small sample size of collected flies, and the limited sampling time. Therefore, further studies must be conducted on a larger scale by sampling more horse farms, using more traps, and having longer sampling periods. Furthermore, molecular studies targeting hemoprotozoan parasites should be performed using both horse blood and biting flies collected from farms to increase the reliability of the use of *Stomoxys* and tabanid flies as markers for the distribution of hemoprotozoan parasites in the study areas.

## Conclusion

To the best of our knowledge, this is the first study on the species composition of *Stomoxys* and tabanid flies and documentation of *T. sinensis* DNA in *S. calcitrans*, *S. sitiens*, *A. cryptotaxis*, and *T. megalops* collected from horse farms, which causes bovine theileriosis. This is the first documented report of *Colpodella* spp. DNA in *S. indicus*. This suggests that these flies can be used as markers for monitoring the circulation of hemoprotozoan parasites in vulnerable areas, leading to effective vector control and disease management in animals.

## Authors’ Contributions

TP: Performed the experiments, analyzed the data, interpreted the results, and drafted the manuscript. PF: Performed the experiments, interpreted the results, and contributed to the writing of the manuscript. SU: Collected fly samples, identified fly samples, and performed the experiments. JA: Interpreted the results and contributed to the writing and revision of the manuscript. NS: Designed and supervised the study, identified fly samples, interpreted the results and drafted and revised the manuscript. All authors have read, reviewed, and approved the final manuscript.

## References

[1] Baldacchino F, Muenworn V, Desquesnes M, Desoli F, Charoenviriyaphap T, Duvallet G (2013). Transmission of pathogens by *Stomoxys* flies (Diptera, Muscidae):A review. Parasite.

[2] Baldacchino F, Desquesnes M, Mihok S, Foil L.D, Duvallet G, Jittapalapong S (2014). Tabanids:Neglected subjects of research, but important vectors of disease agents!. Infect. Genet. Evol.

[3] Taioe M.O, Motloang M.Y, Namangala B, Chota A, Molefe N.I, Musinguzi S.P, Suganuma K, Hayes P, Tsilo T.J, Chainey J, Inoue N, Thekisoe O.M.M (2017). Characterization of tabanid flies (Diptera:Tabanidae) in South Africa and Zambia and detection of protozoan parasites they are harbouring. Parasitology.

[4] Odeniran P.O, Macleod E.T, Ademola I.O, Welburn S.C (2019). Molecular identification of bloodmeal sources and trypanosomes in *Glossina* spp, *Tabanus* spp. and *Stomoxys* spp. Trapped on cattle farm settlements in southwest Nigeria. Med. Vet. Entomol.

[5] Hornok S, Takács N, Szekeres S, Szőke K, Kontschán J, Horváth G, Sugár L (2020). DNA of *Theileria orientalis*, *T. equi* and *T. capreoli* in stable flies (*Stomoxys calcitrans*). Parasit. Vectors.

[6] Williams P (1960). Studies on Ethiopian *Chrysop*s as possible vectors of loiasis. II. Chrysops silacea Austen and human loiasis. Ann. Trop. Med. Parasitol.

[7] Traversa D, Otranto D, Iorio R, Carluccio A, Contri A, Paoletti B, Bartolini R, Giangaspero A (2008). Identification of the intermediate hosts of *Habronema microstoma* and *Habronema muscae* under field conditions. Med. Vet. Entomol.

[8] Ganyukova A.I, Zolotarev A.V, Malysheva M.N, Frolov A.O (2018). First record of *Trypanosoma theileri*-like flagellates in horseflies from Northwest Russia. Protistology.

[9] Kamyingkird K, Yangtara S, Desquesnes M, Cao S, Adjou Moumouni P.K, Jittapalapong S, Nimsupan B, Terkawi M.A, Masatani T, Nishikawa Y, Igarashi I, Xuan X (2014). Seroprevalence of *Babesia caballi* and *Theileria equi* in horses and mules from Northern Thailand. J. Protozool. Res.

[10] Ereqat S, Nasereddin A, Al-Jawabreh A, Al-Jawabreh H, Al-Laham N, Abdeen Z (2020). Prevalence of *Trypanosoma evansi* in livestock in Palestine. Parasit. Vectors.

[11] Rakwong P, Keawchana N, Ngasaman R, Kamyingkird K (2021). *Theileria* infection in bullfighting cattle in Thailand. Vet. World.

[12] Hossain M.J, Raut S, Singh R.P, Mishra P, Hossain M.S, Dey A.R, Kabir A, Anisuzzaman Talukder M.H, Shahiduzzaman M (2023). Molecular detection of *Babesia* and *Theileria* from crossbred cattle in Sirajganj and Rangpur districts of Bangladesh. Vet. Med. Sci.

[13] Changbunjong T, Sedwisi P, Weluwanarak T, Nitiyamatawat E, Sariwongchan R, Chareonviriyaphap T (2018). Species diversity and abundance of *Tabanus* spp. (Diptera:Tabanidae) in different habitats of Thailand. J. Asia Pac. Entomol.

[14] Lorn S, Ratisupakorn S, Duvallet G, Chareonviriyaphap T, Tainchum K (2020). Species composition and abundance of *Stomoxys* spp. (Diptera:Muscidae) in Peninsular Thailand. J. Med. Entomol.

[15] Mantiantipan T, Chibangyang N, Weluwanarak T, Sedwisai P, Changbunjong T (2014). A survey of *Stomoxys* spp. (Diptera:Muscidae) at horse stable of faculty of veterinary science, Mahidol University, Nakhon Pathom Province. J. Appl. Anim. Sci.

[16] Poolkhetkit S, Chowattanapon W, Sungpradit S (2015). Molecular detection of blood protozoa in ticks collected from cattle in the buffer zone of Sai Yok National Park, Thailand. Thai J. Vet. Med.

[17] Jirapattharasate C, Adjou Moumouni P.F, Cao S, Iguchi A, Liu M, Wang G, Zhou M, Vudriko P, Efstratiou A, Changbunjong T, Sungpradit S, Ratanakorn P, Moonarmart W, Sedwisai P, Weluwanarak T, Wongsawang W, Suzuki H, Xuan X (2017). Molecular detection and genetic diversity of bovine *Babesia* spp., *Theileria orientalis*, and *Anaplasma marginale* in beef cattle in Thailand. Parasitol. Res.

[18] Changbunjong T, Sungpradit S, Kanthasaewee O, Sedwisai P, Tangsudjai S, Ruangsittichai J (2016). Molecular detection of *Theileria* and *Babesia* in a diversity of Stomoxyini flies (Diptera:Muscidae) from Khao Yai National Park, Thailand. Thai J. Vet. Med.

[19] Jirapattharasate C, Changbunjong T, Sedwisai P, Weluwanarak T (2018). Molecular detection of piroplasms in haematophagus flies in the Nakhon Pathom and Kanchanaburi Provinces, Thailand. Vet. Integr Sci.

[20] Sontigun N, Boonhoh W, Phetcharat Y, Wongtawan T (2022). First study on molecular detection of hemopathogens in tabanid flies (Diptera:Tabanidae) and cattle in Southern Thailand. Vet.World.

[21] Mihok S (2002). The development of a multipurpose trap (the Nzi) for tsetse and other biting flies. Bull. Entomol. Res.

[22] Burton J.J.S (1978). Tabanini of Thailand above the Isthmus of Kra (Diptera:Tabanidae).

[23] Tumrasvin W, Shinonaga S (1978). Studies on medically important flies in Thailand. V. On 32 species belonging to the subfamilies Muscinae and Stomoxyinae including the taxonomic keys (Diptera:Muscidae). Bull. Tokyo Med. Dent. Univ.

[24] Burger J.F, Chainey J.E (2000). Revision of the oriental and Australasian species of *Chrysops* (Diptera:Tabanidae). Invertebr. Taxon.

[25] Kledmanee K, Suwanpakdee S, Krajangwong S, Chatsiriwech J, Suksai P, Suwannachat P, Sariya L, Buddhirongawatr R, Charoonrut P, Chaichoun K (2009). Development of multiplex polymerase chain reaction for detection of *Ehrlichia canis*, *Babesia* spp and *Hepatozoon canis* in canine blood. Southeast Asian J. Trop. Med. Public Health.

[26] Njiru Z.K, Constantine C.C, Guya S, Crowther J, Kiragu J.M, Thompson R.C, Dávila A.M (2005). The use of ITS1 rDNA PCR in detecting pathogenic African trypanosomes. Parasitol. Res.

[27] Hall T.A (1999). BioEdit:A user-friendly biological sequence alignment editor and analysis program for Windows 95/98/NT. Nucl. Acids. Symp. Ser.

[28] Tamura K, Nei M (1993). Estimation of the number of nucleotide substitutions in the control region of mitochondrial DNA in humans and chimpanzees. Mol. Biol. Evol.

[29] Kumar S, Stecher G, Li M, Knyaz C, Tamura -K (2018). MEGA X:Molecular Evolutionary Genetics Analysis across computing platforms. Mol. Biol. Evol.

[30] Muenworn V, Duvallet G, Thainchum K, Tuntakom S, Tanasilchayakul S, Prabaripai A, Akratanakul P, Sukonthabhirom S, Chareonviriyaphap T (2010). Geographic distribution of stomoxyine flies (Diptera:Muscidae) and diurnal activity of *Stomoxys calcitrans* in Thailand. J. Med. Entomol.

[31] Changbunjong T, Weluwanarak T, Ratanakorn P, Maneeon P, Ganpanakngan M, Apiwathnasorn C, Sungvornyothin S, Sriwichai P, Sumruayphol S, Ruangsittichai J (2012). Distribution and abundance of Stomoxyini flies (Diptera:Muscidae) in Thailand. Southeast Asian J. Trop. Med. Public Health.

[32] Bai Q, Liu G, Yin H, Zhao Q, Liu D, Ren J (2002). *Theileria sinensis* sp nov:A new species of bovine *Theileria*-molecular taxonomic studies. Xu Mu Shou Yi Xue Bao.

[33] Bursakov S.A, Kovalchuk S.N (2019). Co-infection with tick-borne disease agents in cattle in Russia. Ticks Tick Borne Dis.

[34] Agina O.A, Shaari M.R, Isa N.M.M, Ajat M, Zamri-Saad M, Mazlan M, Muhamad A.S, Kassim A.A, Ha L.C, Rusli F.H, Masaud D, Hamzah H (2021). Molecular detection of *Theileria* species, *Anaplasma* species, *Candidatus*
*Mycoplasma haemobos*, *Trypanosoma evansi* and first evidence of *Theileria sinensis*-associated bovine anaemia in crossbred Kedah-Kelantan x Brahman cattle. BMC Vet. Res.

[35] Chen Y, Chen Y.Y, Liu G, Lyu C, Hu Y, An Q, Qiu H.Y, Zhao Q, Wang C.R (2022). Prevalence of *Theileria* in cattle in China:A systematic review and meta-analysis. Microb. Pathog.

[36] Kuvardina O.N, Leander B.S, Aleshin V.V, Myl'nikov A.P, Keeling P.J, Simdyanov T.G (2002). The phylogeny of colpodellids (Alveolata) using small subunit rRNA gene sequences suggests they are the free-living sister group to apicomplexans. J. Eukaryot. Microbiol.

[37] Yuan C.L, Keeling P.J, Krause P.J, Horak A, Bent S, Rollend L, Hua X.G (2012). *Colpodella* spp.-like parasite infection in woman, China. Emerg. Infect. Dis.

[38] Jiang J.F, Jiang R.R, Chang Q.C, Zheng Y.C, Jiang B.G, Sun Y, Jia N, Wei R, Liu H.B, Huo Q.B, Wang H, von Fricken M.E, Cao W.C (2018). Potential novel tick-borne *Colpodella* species parasite infection in patient with neurological symptoms. PLoS Negl. Trop. Dis.

[39] Matsimbe A.M, Magaia V, Sanches G.S, Neves L, Noormahomed E, Antunes S, Domingos A (2017). Molecular detection of pathogens in ticks infesting cattle in Nampula Province, Mozambique. Exp. Appl. Acarol.

[40] Chiu H.C, Sun X, Bao Y, Fu W, Lin K, Chen T, Zheng C, Li S, Chen W, Huang C (2022). Molecular identification of *Colpodella* spp. of South China tiger *Panthera tigris amoyensis* (Hilzheimer) in the Meihua Mountains, Fujian, China. Folia Parasitol (Praha).

[41] Xu M, Hu Y, Qiu H, Wang J, Jiang J (2022). *Colpodella* spp. (Phylum Apicomplexa) identified in horses shed light on its potential transmission and zoonotic pathogenicity. Front. Microbiol.

